# The TGFBR1*6A allele is not associated with susceptibility to colorectal cancer in a Spanish population: a case-control study

**DOI:** 10.1186/1471-2407-9-193

**Published:** 2009-06-18

**Authors:** Adela Castillejo, Trinidad Mata-Balaguer, Paola Montenegro, Enrique Ochoa, Rafael Lázaro, Ana Martínez-Cantó, María-Isabel Castillejo, Carla Guarinos, Víctor-Manuel Barberá, Carmen Guillén-Ponce, Alfredo Carrato, José-Luís Soto

**Affiliations:** 1 Molecular Oncology Group, Elche University Hospital, Camino Almazara 11, 03203 Elche, Spain; 2 Genetic Counseling in Cancer Unit, Elche University Hospital, Camino Almazara 11, 03203 Elche, Spain; 3 Molecular Biopathology Department. Castellon Provincial Hospital. Avenida Doctor Clara 19, 12002 Castellon de La Plana, Spain; 4 Pathology Department, La Plana Hospital, Partida Carinyena Km 0.5, 12540 Vila-real. Spain

## Abstract

**Background:**

TGF-β receptor type I is a mediator of growth inhibitory signals. TGFBR1*6A (rs11466445) is a common polymorphic variant of the TGF-β receptor I gene and has been associated with tumour susceptibility. Nevertheless, the role of this polymorphism as a risk factor for colorectal cancer is controversial. The aim of this study was to assess the association between TGFBR1*6A and colorectal cancer, age, sex, tumour location and tumour stage in a Spanish population.

**Methods:**

The case-control study involved 800 Spanish subjects: 400 sporadic colorectal cancer patients and 400 age-, sex-, and ethnic-matched controls. The odds ratio (OR) and 95% confidence interval (95% CI) for the TGFBR1*6A polymorphism were calculated using unconditional logistic regression adjusted for age and sex. Analysis of somatic mutations at the GCG repeat of *TGFBR1 *exon 1 and germline allele-specific expression were also conducted to obtain further information on the contribution of the TGFBR1*6A allele to CRC susceptibility.

**Results:**

There was no statistically significant association between the TGFBR1*6A allele and CRC (p > 0.05). The OR was 1.147 (95% CI: 0.799–1.647) for carriers of the TGFBR1*6A allele and 0.878 (95% CI: 0.306–2.520) for homozygous TGFBR1*6A individuals compared with the reference. The frequency of the polymorphism was not affected by age, sex or tumour stage. The TGFBR1*6A allele was more prevalent among colon tumour patients than among rectal tumour patients. Tumour somatic mutations were found in only two of 69 cases (2.9%). Both cases involved a GCG deletion that changed genotype 9A/9A in normal DNA to genotype 9A/8A. Interestingly, these two tumours were positive for microsatellite instability, suggesting that these mutations originated because of a deficient DNA mismatch repair system.

Allele-specific expression of the *9A *allele was detected in seven of the 14 heterozygous 9A/6A tumour cases. This could have been caused by linkage disequilibrium of the TGFBR1*6A allele with mutations that cause allele-specific expression, as was recently suggested.

**Conclusion:**

Our results suggest that the TGFBR1*6A allele does not confer an increased risk of colorectal cancer in the Spanish population.

## Background

Colorectal cancer (CRC) is the main cause of cancer-related deaths in Spain. Although environmental and genetic factors associated with cancer have been described, current knowledge of the genetic basis of colorectal cancer is limited. About 5% of colorectal cancer cases are associated with germline mutations of genes such as *APC*, DNA mismatch repair genes, *MYH*, *SMAD4 *and *BMPR1A/ALK3 *[1]. The heritability component of the remaining 95% of cases is unknown.

Low-penetrance cancer susceptibility alleles may increase the risk of cancer in a manner similar to that of pathogenic germline mutations in hereditary cancer genes. Transforming growth factor beta (TGF-β) is one of the most potent inhibitors of cell growth. TGF-β ligands interact with the type II receptor and then bind to the type I receptor, sending a signal to the nucleus through *SMAD *proteins [2]. Inhibition of TGF function causes unrestricted cell growth because of a lack of growth inhibition. Thus, germline mutations of *TGFBR2 *and *SMAD4 *may predispose to the development of hereditary non-polyposis colorectal cancer and juvenile polyposis, respectively [3].

Recently, it has been suggested that a mouse model carrying a germline Tgfbr1 haploinsufficiency has constitutively reduced TGF-beta signalling that significantly enhances the development of colorectal cancer [4].

Polymorphisms of various genes associated with the TGF-β pathway have been described as cancer risk factors. TGFBR1*6A (rs11466445) is a common polymorphic variant of the TGF-β receptor I gene. It has been suggested that the TGFBR1*6A allele is a low-penetrance susceptibility factor for colorectal cancer [5-7]. This variant of *TGFBR1 *is generated by deletion of three *GCG *triplets that code for three alanine molecules within a nine alanine (*9A) stretch sequence at exon 1. The TGFBR1*6A allele encodes a type I receptor with reduced growth-inhibitory signalling activity [5]. Two meta-analyses, which included 12 case-control studies, have shown that TGFBR1*6A is a candidate tumour-susceptibility allele, is present in 13.7% of the general population and increases the risk of cancer by approximately 24% [6,7]. Somatic acquisition of this trinucleotide variant has also been reported in colon cancer, supporting its role as a cancer risk allele [8]. Moreover, it has been proposed that TGFBR1*6A is responsible for a proportion of patients with hereditary non-polyposis colorectal cancer syndrome [9]. In contrast, based on their case-control study and another meta-analysis [10], Skoglund *et al. *[10] suggested that this variant is not associated with colorectal cancer. Similarly, others have reported that there is no association between the TGFBR1*6A allele and prostate [11], lung [12] or bladder [13] cancer. On the other hand, Valle *et al. *[14] recently suggested that germline allele-specific expression (ASE) of the *TGFBR1 *gene is dominantly inherited, segregates in families and occurs in sporadic CRC cases. This ASE confers a substantially increased risk of CRC (OR: 8.7; 95% CI: 2.6–29.1). Moreover, the authors claim that the TGFBR1*6A allele is probably in linkage disequilibrium with one of the putative mutations that causes ASE, but TGFBR1*6A per se does not cause ASE. The causative germline changes have not been identified.

Because of this controversy, we conducted a case-control study of the TGFBR1*9A/6A polymorphism, including mutational and ASE analyses, to clarify the effect of the TGFBR1*6A polymorphism on the risk of colorectal cancer in our population.

## Methods

### Subjects

A total of 400 sporadic CRC cases and 400 controls from the Elche University Hospital and Castellon Provincial Hospital tissue banks were analysed. Written consent to be included in the tissue banks was obtained from each patient. Patients diagnosed with a familial cancer syndrome were excluded.

This was a hospital-based case-control study. Controls with no personal history of cancer, selected with diagnoses considered unrelated to the exposures of interest, were selected from the same hospitals and matched with cases for age, sex and race/ethnicity. The study was approved by the ethical committees of the Elche University Hospital and the Castellon Provincial Hospital.

The median age at diagnosis of CRC was 70 years (range 22–93 years) and that of the controls was 72 years (range 23–98 years). The sex distribution was 41.8% female (n = 167) and 58.2% male (n = 233) for colorectal cancer patients and 53.2% female (n = 213) and 46.8% male (n = 187) for the controls.

Of the CRC cases, 24% had proximal colon tumours, 33.75% had distal colon tumours, 9.25% had sigma-rectum tumours and 25% had rectal tumours. In 8% of cases, the location of the tumour was unknown. Cases were classified according to tumour stage as low stage (stages I and II: 78.5% of cases) or high stage (stages III and IV: 21.5% of cases) (Table 1).

**Table 1:** TGFBR1 rs11466445 genotype frequency according to tumour location and stage (UK: unknown)

Specimens	n	9/9	6/9	6/6	*p*
**Tumour location**					
Proximal colon	93	77	15	1	
Distal colon	135	99	34	2	*p = 0.109*
Rectum	100	88	10	2	***p = 0.024***
Rectum sigma	37	32	4	1	
UK	35				
**Stage**					
I-II (low)	139	108	28	3	
III-IV (high)	38	31	7	0	*p = 0.66*
UK	223				

### DNA and RNA samples

DNA for the case-control study: Control DNA was obtained from peripheral blood samples (n = 400). DNA was isolated from the non-tumour colorectal tissue (n = 400) of cases after mechanical homogenization *(TissueLyser, Qiagen, Valencia, CA)*. DNA isolation was performed using the *EZ1 DNA Tissue kit *and the *EZ1 BioRobot (Qiagen, Valencia, CA) *according to the manufacturers' instructions.

DNA for the *TGFBR1 *exon 1 tumour mutation: Tumour tissue DNA was isolated after mechanical homogenization of colorectal tumour tissues (n = 69) as described previously.

RNA extraction: RNA was isolated from normal colorectal tissue samples from 14 heterozygous 9A/6A CRC patients after mechanical homogenization using the *EZ1 RNA Tissue kit *and the *EZ1 BioRobot (Qiagen, Valencia, CA)*.

cDNA synthesis: A retrotranscription reaction was performed using *Reverse Transcription Reagents*, random primers (*Applied Biosystems, Foster City, CA*) and 200–1000 ng of RNA.

### Analysis of the TGFBR1 exon 1 polymorphism, TGFBR1*9A/6A

Genotyping of the TGFBR1 exon 1 polymorphism was conducted in the case-control study and used for the somatic mutation and ASE analyses. Genotype was assessed using PCR amplification and capillary electrophoresis. The PCR primers were *CCACAGGCGGTGGCGGCGGGACCATG *(5'-labelled with 6-FAM) for *TGFBR1F *and *CGTCGCCCCCGGGAGCAGCGCCGC *for *TGFBR1R*. PCR amplification was done using the Amplitaq Gold PCR Master Mix 2× in a total volume of 25 μl containing 1 M betaine (Sigma Aldrich). The PCR was run for 35 cycles at 94°C for 30 s and 72°C for 90 s followed by a 10 min final extension at 72°C. PCR products and size standards (*Genescan 500 ROX size standard; Applied Biosystems, Foster City, CA*) were diluted in HiDi Formamide (*Applied Biosystems, Foster City, CA*) and then resolved by capillary electrophoresis (*ABI 3100 Avant, Applied Biosystems, Foster City, CA*) using POP6 as polymer. GeneScan software was used for analysis of PCR fragments (*Applied Biosystems, Foster City, CA*). Representative cases for each genotype were sequenced directly to confirm allele sizes (data not shown). A PCR product size of 119 bp corresponded to the most common allele, *9A, whereas a product size of 110 bp corresponded to the *6A allele.

### Microsatellite instability (MSI) status

A subset of colorectal tumour DNAs from 120 patients was screened for MSI status using five mononucleotide markers (BAT26, BAT25, NR21, NR24 and NR27) and multiplex PCR as previously described by Buhard *et al*. [15].

### Allele-specific expression of heterozygous *6A/*9A individuals

Fourteen heterozygous *9A/6A *CRC cases were included in the ASE analysis. TGFBR1 exon 1 amplicons generated from normal genomic DNA and corresponding normal cDNA were run in parallel. The ASE ratio was calculated by normalizing the ratio between the peak areas of the two alleles in cDNA with the same parameters in genomic DNA. A threshold ratio of a 33% difference was used to define ASE-positive cases [14].

### Statistical analysis

The Hardy-Weinberg equilibrium was checked among the control and case populations. In the case-control study, we estimated the odds ratio (OR) and 95% confidence interval (95% CI) for the TGFBR1*6A polymorphism using unconditional logistic regression adjusted for age and sex. We analysed for potential effect modification by age using an analysis stratified according to median age at diagnosis (≤ 70 years or >70 years). A χ^2 ^test was used to evaluate differences in TGFBR1*6A carrier frequencies between the tumour and control groups and to analyse the association between the polymorphism and the clinical and pathological factors. A probability level of < 0.05 was considered significant.

## Results

### Case-control study

The genotype distribution in the case and control populations did not deviate significantly from that expected for a population in Hardy-Weinberg equilibrium (p = 0.295 for cases and p = 0.033 for controls). The allele frequencies for the TGFBR1*6A allele were 0.101 and 0.092 for cases and controls, respectively. Genotype distributions for cases and controls are presented in Table 2. No significant association was observed between the TGFBR1*6A allele and colorectal cancer incidence in our population (p = 0.517). The crude ORs were 1.147 (95% CI: 0.799–1.647) for carriers of the TGFBR1*6A allele and 0.878 (95% CI: 0.306–2.520) for TGFBR1*6A homozygous individuals compared with the reference (homozygous TGFBR1*9A).

**Table 2:** TGFBR1 rs11466445 genotype frequency in cases and controls in Spain

TGFBR1 exon 1 genotype	Cases (n = 400) number (%)	Controls (n = 400) number (%)	OR (95%CI)	*p*
9A/9A,	325 (81.25)	333 (83.25)	1 (ref.)	
6A/9A,	69 (17.25)	60 (15)	1.178 (0.808–1.718)	*0.441*
6A/6A,	6 (1.5)	7 (1.75)	0.878 (0.306–2.520)	*1*
6A/*	75 (18.75)	67 (16.75)	1.147 (0.799–1.647)	*0.517*

There was no statistically significant association when the OR was adjusted for age and sex. When we analysed the cases according to clinical stage, we detected no significant difference in the TGFBR1*6A allele distribution between low and high stages (p = 0.66) (Table 1). On the other hand, TGFBR1*6A allele carriers were more prevalent among colon (proximal plus distal) cancer patients than rectal cancer patients (p = 0.024) (Table 1).

### Frameshift somatic mutation at TGFBR1 exon 1

**Figure 1:**
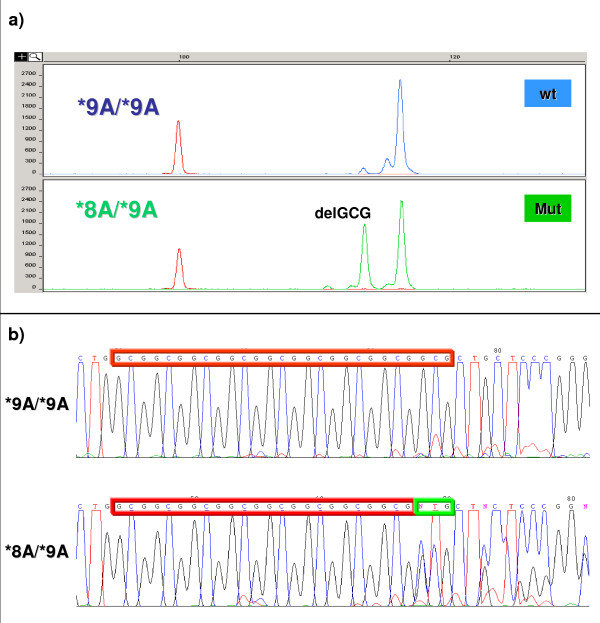
**Somatic mutation at exon 1 of the *TGFBR1 *gene in MSI tumours**. A *GCG *deletion of the triplet repeat was found in two independent cases: a) a PCR fragment resolved by capillary electrophoresis, b) DNA sequence from a wild-type case (*9A/*9A) and a mutated case (*8A/*9A).

First, we screened colorectal tumour DNA from 120 patients for MSI status. We found evidence of MSI in 14 cases (14/120, 11.67%). Two of 14 MSI tumours (14.29%) had a deletion of a *GCG *triplet. All 14 MSI cases were homozygous for the TGFBR1*9A allele. Both mutated cases were genotyped as heterozygous *9A/8A*. These results were confirmed by direct sequencing (Figure 1). To determine whether these somatic mutations are specific for MSI tumours, we analysed tumour DNA from 55 cases that were homozygous for the TGFBR1*9A allele and were MSI-negative. None of these 55 cases had a mutation of this *GCG *repeat (p = 0.039; two-tailed chi square test).

### Allele-specific expression in heterozygous *6A/*9A individuals

**Figure 2:**
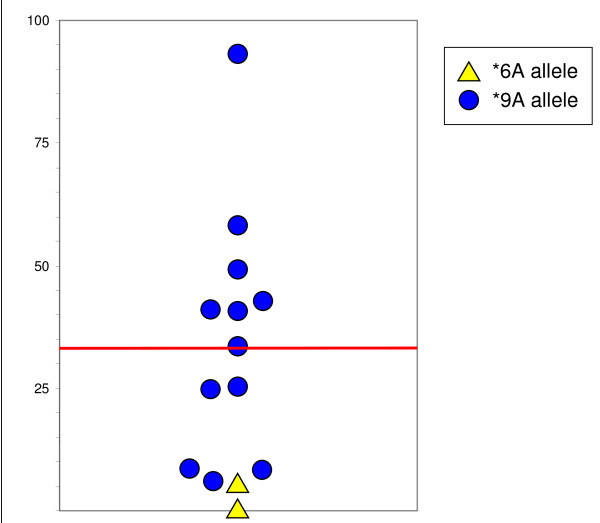
Allele-specific expression of heterozygous *6A/*9A individuals. The over-expressed allele and the ratio of over-expression are presented. The ASE threshold was defined as 33%.

In the genotyping experiments, comparisons between peak areas generated from heterozygous individuals from gDNA and cDNA enabled us to obtain information about the principally expressed allele and the relative level of its expression. Seven of 14 cases had differences greater than 33% and were considered ASE-positive. Interestingly, all seven cases with ASE over-expressed the TGFBR1*9A allele (Figure 2).

## Discussion

There is evidence that common variants of the TGF-β pathway alter TGF-β signalling and modify cancer risk. The TGFBR1*6A variant has been associated with breast, ovarian and haematological malignancies [6,9]. Recent studies suggest that the association of the TGFBR1*6A allele with colon cancer is either weak (OR: 1.2; 95% CI: 1.01–1.43) [7] or of borderline significance (OR: 1.13; 95% CI: 0.98–1.30) [10].

In association studies, contradictory conclusions may arise from population stratification or inappropriate sample size. We determined *a priori *that our study with 400 sporadic CRC cases and 400 matched controls would enable us to detect an OR of 2.0 for *9A/6A *heterozygous individuals (assuming a frequency of 0.15 in controls) and an OR of 3.0 for *6A/6A *homozygous individuals (assuming a frequency of 0.0175 in controls) with 80% power (two-sided test, alpha level = 5%).

Our results did not show a statistically significant association (p = 0.517; OR: 1.147; 95% CI: 0.799–1.647), which is in agreement with Skoglund *et al. *[10]. To our knowledge, the only previous study of this polymorphism in a Spanish population is one that was included in the meta-analysis of Pasche *et al. *[7]. That case-control study was performed with 298 controls, 237 sporadic CRC cases and 275 sporadic breast cancers. When data for both types of tumour were pooled, a significant increase in cancer risk was associated with TGFBR1*6A carriers (OR: 1.60; 95% CI: 1.10–2.31). The study did not provide information specifically about CRC or about sex matching between the groups. Zhang *et al. *(2005) suggested that the 12 case-control studies of Pasche [7] had marginally significant heterogeneity when pooled and questioned the association between TGFBR1*6A and increased risk of cancer [16]. Similar doubts have been raised in various studies involving different types of tumours [11-13] and in a meta-analysis that included eight case-control studies [10].

There was little information on which to base the sample size for our study because information about this polymorphism in our population is limited and contradictory results were reported for another population. The results show that our study was under-powered for detecting weak associations between the TGFBR1*6A allele and CRC. The data indicate that a sample size of 10,000 cases and 10,000 controls is necessary to detect an OR of 1.15 with 80% power using a two-sided test with an alpha level of 5%.

In the present work, age and sex adjustment did have any effect on the risk conferred by the TGFBR1*6A allele. Previous case-control studies provide little information in this regard.

Interestingly, there was a significant difference in the frequency of the TGFBR1*6A allele between colon and rectal cancer cases. Moreover, when only colon cancer cases where considered, there was a difference of borderline significance for TGFBR1*6A carriers (p = 0.071) with an OR = 1.47 (95% CI: 0.979–2.203). No data are available regarding the risk of colon versus rectal cancer for the TGFBR1*6A allele because data from colorectal and colon cancer studies were pooled in the meta-analyses. Further studies with larger samples are needed to determine if the variant allele is associated specifically with colon cancer but not with rectal cancer. If confirmed, this may help explain the contradictory results in the literature.

Because of the limitation that sample size exerts on association studies, we attempted to extract extra information from our data to elucidate the susceptibility effect of the minor allele of this polymorphism. First, we wanted to know if the *GCG *repeat is a specific target for frameshift mutations in MSI colorectal tumours, and in the event, if somatic acquisition of TGFBR1*6A allele occurs in these MSI tumours, similar to metastatic CRC [8]. The underlying hypothesis was that if the minor allele in the germline predisposes to CRC, it might be prevalent in the tumours themselves through somatic acquisition. Our results show that the *GCG *repeat at exon 1 of the *TGFBR1 *gene is a specific target for MSI tumours (p = 0.039), but the frequency of mutations is low (2/14, 14.29%) [17]. Nevertheless, there was no somatic acquisition of the TGFBR1*6A allele. Both mutated cases underwent the deletion of just one *GCG *triplet. No previous studies have documented the association between this polymorphism and MSI status. Unfortunately, our results did not enable us to clarify the role of the TGFBR1*6A allele in CRC risk.

An additional strategy was used to address the role of the minor allele for this polymorphism in CRC. We tested whether gene expression level differed between the alleles of the polymorphism because previously reported data from *in vitro *models shows that growth inhibitory signalling activity is low for the type I receptor coded by the TGFBR1*6A allele [5]. We hypothesized that if predominant expression of the TGFBR1*6A allele exists in the *6A/9A *heterozygous individual, global functional reduction of the pathway would be evident and would increase the risk for CRC. Surprisingly, the results of the ASE study were opposite to what we had expected. The proportion of ASE positive cases was high (7/14), and there was predominant expression of the TGFBR1*9A allele in all seven ASE positive cases. The correct interpretation of our results became evident after the recent publication of the study of Valle *et al. *[14]. The authors concluded that ASE of *TGFBR1 *is a major contributor to genetic predisposition for CRC (OR: 8.7; 95% CI: 2.6–29.1). They were unable to determine the causative mechanism of the ASE but the haplotype data suggested that ancestral mutations were implicated. One of the putative mutations that causes ASE is probably in linkage disequilibrium with the TGFBR1*6A allele but is not itself causative of ASE. Our results are in agreement with those of Valle *et al. *in that about 50% of *6A/9A *heterozygous individuals have ASE and the *6A *allele is expressed at a low level in all cases. The linkage disequilibrium between ASE and the TGFBR1*6A allele could explain the contradictory results in the literature on the association between this polymorphism and CRC.

## Conclusion

The case-control study did not show a statistical association between the TGFBR1*6A allele and CRC, age, sex or tumour stage. The differences in haplotype frequencies between colon and rectal tumour patients should be confirmed by larger series. The somatic mutations that affected the *GCG *repeat in this study are a consequence of deficient MMR function. The ASE results suggest that there is linkage disequilibrium between the TGFBR1*6A allele and the mutations that cause ASE. Taken together, our results strongly suggest that the TGFBR1*6A allele does not confer an increased risk of colorectal cancer in the Spanish population.

## Competing interests

The authors declare that they have no competing interests.

## Authors' contributions

AC participated in the design and coordination of the study, in the molecular genetic studies and helped to draft the manuscript. TM-B, AM-C, M-IC, CG and V-MB participated in the molecular genetic studies and helped to draft the manuscript. PM participated in the design of the study, performed the statistical analysis and helped to draft the manuscript. EO and RL participated in the biobanking of samples and helped to draft the manuscript. CG-P and AC participated in the design of the study and performed the statistical analysis. J-LS conceived the study, participated in its design and coordination and drafted the manuscript. All authors read and approved the final manuscript.

## Pre-publication history

The pre-publication history for this paper can be accessed here:

http://www.biomedcentral.com/1471-2407/9/193/prepub
